# Psychiatric Disorders During Pregnancy and Postpartum Period: A Guide For Perinatal Interview

**DOI:** 10.5152/eurasianjmed.2023.23329

**Published:** 2023-12-01

**Authors:** Esra Yazıcı, Sema Şahin

**Affiliations:** Department of Psychiatry, Sakarya University Faculty of Medicine, Sakarya, Türkiye

**Keywords:** Antidepressants, antipsychotics, postpartum, pregnancy

## Abstract

Pregnancy and the 1-year period after birth are special periods for the follow-up and treatment of psychiatric diseases. Many diagnoses, including depressive disorders, anxiety disorders, bipolar disorder, and psychotic disorders, appear in these periods.

The treatment process starts with a perinatal interview that contains a careful biopsychosocial examination of the patient and shared decision-making with a realistic assessment of the risks and benefits of treatment options.

Perinatal mental problems can harm the physical and emotional development of the baby as well as the health of the mother, and these problems bring a significant burden of disease globally.

There is a massive amount of literature that suggests using most psychotropic medicines is safer than the harms of an untreated mental illness.

## Introduction

Psychiatric disorders are common and affect individuals, families, and communities arround the world and in our country. Women, in particular, bear a high burden of high psychiatric disorder prevalence.^1^ Women are at risk for psychiatric disorders throughout their lives, and 1 of the periods when this risk increases is the reproductive age, which naturally accompanies a woman’s life. Pregnancy and the 1-year period after birth are special periods for the follow-up and treatment of psychiatric diseases. Many diagnoses, including depressive disorders, anxiety disorders, bipolar disorder, and psychotic disorders, can appear during these periods. These conditions can occur before, during, and after pregnancy.^2^ Women experience hormonal fluctuations during their menstrual cycle, premenstrual syndrome (PMS), menopause, pregnancy, and the postpartum period. Sometimes, these fluctuations can be associated with psychiatric symptoms.^3-5^ Approximately 1 in 5 women develops a mental illness.^6,7^

Treating psychiatric disorders during the perinatal period is important for the mental and physical health of both the mother and the baby. For this reason, the physician who meets with the pregnant woman or mother should be able to make a psychological evaluation, provide information, and provide treatment opportunities with both pharmacotherapy and psychosocial interventions.^7,8^ Psychotherapy and psychosocial interventions are the preferred treatment methods for people with mild and moderate mental illness, but planning and decision-making should be prioritized by discussing them with the patient in terms of accessibility and feasibility.^9^

During pregnancy and the postpartum period, women may also need to use medication due to psychiatric disorders. In a study conducted at a university hospital in the east of Turkey, it was determined that psychotropic drug use during pregnancy was highest in the schizophrenia group, moderate in the bipolar group, and lowest in the depressive disorder group. They found no significant difference between patient groups in terms of psychotropic drug use during breastfeeding.^1^ Treatment of mental disorders is important for women, babies, and communities. This guide aims to provide you with information on the identification, evaluation, and management of perinatal mental disorders (from pregnancy to 1 year postpartum).

## Psychiatric Disorders in the Perinatal Period

### Depressive Disorders

The Diagnostic and Statistical Manual of Mental Disorders (DSM-5-TR) defines perinatal depression as a major depressive disorder with a time-of-onset marker. Specifically, this includes the onset of depressive symptoms during pregnancy or up to 4 weeks postpartum.^10^

Depression during pregnancy is a common mental disorder that affects the health of both the mother and the baby. The prevalence of depression and depressive symptoms during pregnancy varies between 12% and 36% in terms of general prevalence.^11^ When pregnancy periods are examined separately, it has been shown that the prevalence of depression is as high as 7.4% in the first trimester, 12% in the second and third trimesters, and even higher in the first year after birth.^12^ The prevalence of postpartum depression was found to be 14.0% in a large meta-analysis, but this rate varies from country to country (5.0%-26.32%), with higher prevalences being seen mainly in developing countries.^13^

In terms of postpartum depression, having a depressive disorder in the first 3 months of pregnancy, previous mental disorders, somatic disorders, exposure to domestic violence during pregnancy, keeping the baby in an incubator, and not being able to breastfeed are essential risk factors.^14,15^ Additionally, a low education level, young maternal age, having more than 3 children, and high premenstrual symptoms were risk factors for depressive disorders in the non-perinatal reproductive age group.^16,17^

The most critical risk factor for postpartum depression is depression during pregnancy.^14^ In a study conducted by Yazıcı et al, women diagnosed with depression during pregnancy were contacted in the postpartum period. Postpartum depression diagnosis rates and scores of women who were treated for depression during pregnancy and those who were not treated were compared. When the results were examined, it was determined that the depression scores of those who received treatment were lower than before birth, and none of them were diagnosed with postnatal depression. Additionally, in the same study, depression was detected in 92% of those who did not seek treatment, and it was observed that general depression scores in this group increased in the postpartum period compared to the prenatal period.^18^

Another health problem during pregnancy is suicide and suicidal ideation. Suicide is a leading cause of death during the perinatal period in high-income countries (accounting for 5 to 20% of maternal deaths).^19^ In a recent study, the prevalence of suicidal ideation was reported to be 8%, with this rate being 10% in the prenatal period and 7% in the postnatal period.^20^

### Anxiety Disorders

Subtypes of anxiety disorders, according to DSM-5, are generalized anxiety disorder, panic disorder, agoraphobia, separation anxiety disorder, social phobia, selective mutism, and phobia-related disorders.^10^ Anxiety disorders are quite common in the general female population, with 1 in 5 women shown to meet diagnostic criteria for 1 or more disorders.^21^ Anxiety disorders can cause severe functional impairments, and although they are not specific to pregnancy or postpartum, individuals with anxiety are more likely to have suicidal thoughts and attempts than those without anxiety.^22^ Anxiety disorders during pregnancy have been reported to be associated with premature birth and low birth weight, as well as the discomfort caused to the pregnant woman and the decrease in quality of life due to the nature of the disease.^23^

### Bipolar Disorder

The onset of bipolar disorder typically occurs in late adolescence or early adulthood.^24^ Therefore, people with bipolar disorder are generally more likely to be diagnosed with bipolar disorder during their reproductive years. Physicians need to recognize bipolar disorders in obstetrics and gynecology clinics because childbirth is one of the most potent triggers of hypomania or mania episodes.^25^ It is also known that women with bipolar disorder have a much higher risk of experiencing a manic attack in the postpartum period than in any other period of their lives.^26^ In a large-scale meta-analysis, the prevalence of bipolar disorder in women without any known psychiatric disease before the perinatal period was found to be 2.6%, and the prevalence of any mood episode in the bipolar spectrum during pregnancy and the postpartum period was 20.1%. It was observed that 54.9% of women previously diagnosed with bipolar disorder experienced at least 1 mood episode on the bipolar spectrum during the perinatal period.^27^

In bipolar disorder, depressive attacks may also occur, as well as manic attacks. It is important to note that almost a quarter (22.6%) of individuals who screen positive for perinatal depression may have bipolar disorder upon further evaluation.^6^

The recurrence of postpartum bipolar disorder varies by individual but is generally around 35% and is significantly higher in those not treated with pharmacotherapy compared to those who continue treatment.^28^ Discontinuing pharmacotherapy for bipolar disorder during pregnancy or postpartum has a 3-fold increased risk of recurrence of a manic or depressive episode compared to discontinuing pharmacotherapy in non-perinatal women.^29^ Additionally, untreated bipolar disorder patients have a 25-50% increased risk of postpartum psychosis.^30^ Postpartum psychosis is known to increase the risk of suicide and harmful behavior.^31^ Untreated bipolar disorder, in addition to its potential harm to pregnant women, can lead to problems such as fetal growth retardation, premature birth, and negative effects on neurodevelopment.^32^ Therefore, it is crucial to continue effective treatment management and pharmacotherapy for pregnant women with bipolar disorder.

### Psychotic Disorders

Postpartum psychosis often occurs within the context of bipolar disorder and may occur during an episode of mania, depression, or a mixed episode with psychotic features. This patient group, in particular, requires psychiatric monitoring during pregnancy and the postpartum period and should be seen by a psychiatrist.^33^ Although the DSM-5 states that this diagnosis occurs “within the first 4 weeks postpartum,” the typical onset is 3-10 days after birth and can also occur after 4 weeks postpartum.^10^ Symptoms of postpartum psychosis may include agitation, delusions, hallucinations, disorganized thoughts, and disorganized behavior. Patients often have no insight into their symptoms and may experience a significant decline in average daily functioning.^34^ The majority of young women who apply to hospitals with a diagnosis of postpartum psychosis do not have a known psychiatric history and experience their first attacks in the postpartum period.^35^

The partner, family, friends, and other social support systems, mental health professionals, and the birth team should collaborate to coordinate postpartum psychosis prevention planning, including psychopharmacology, observation, social support, and adequate sleep strategies.^36^ Postpartum psychosis is a severe psychiatric emergency that involves the risk of infanticide and suicide. Patients with psychotic symptoms should undergo a comprehensive clinical assessment to determine the need for hospitalization. This assessment should cover the patient’s psychiatric status, physical condition, social situation, and living conditions. The decision of whether or not to hospitalize the patient should be made based on the assessment results.^2^

### Other Psychiatric Disorders

Pregnancy and the postpartum period alone are not protective against any psychiatric disorder, and all psychiatric disorders seen in women can also be seen during this period.

Research shows that the perinatal period is a risk factor for the onset or exacerbation of obsessive-compulsive disorder (OCD), with pregnant and postpartum women having a 1.5-2 times higher risk of OCD compared to the general population.^37^ It has been found that the prevalence of OCD during pregnancy varies between 0.2% and 3.5%, and the prevalence of OCD after birth varies between 2.7% and 9%.^37,38^ Postpartum exacerbation rates of OCD are estimated to be between 25% and 75%.^39,40^ Obsessions about losing the baby are especially common in the postpartum period.^39^ Additionally, it has been observed that in 13-39% of women with OCD, the onset of OCD occurs during pregnancy, especially in the second trimester.^41^

Tobacco, alcohol, and substance use are other problems that should be taken into consideration during pregnancy. Smoking and drug and alcohol use disorders during pregnancy are common, and premature birth, intrauterine growth restriction, and fetal diseases are more common in women who use these substances, especially women who smoke.^42,43,44^

### Perinatal Psychiatric Interview: A Realistic Assessment and Psychoeducation for Risks and Benefits

In the perinatal period, treatment begins with a “perinatal interview,” described by Yazici and Aydin 2021^45^, summarized in 6 steps. It is the first interview by a specialist with a woman in the perinatal period. It contains a specific evaluation of the patient’s psychiatric condition in a biopsychosocial model, and pharmacological and/or psychosocial interventions are planned (Table 1).

### Step 1: Take responsibility for the Treatment

Management of individuals with mental illness, especially pregnant women, is a challenging situation for physicians and other healthcare professionals.^46^ In interviews with psychiatry professionals, it has been reported that they tend to avoid starting drug treatment during pregnancy, end ongoing drug treatment, look for an alternative to drug treatment, switch to (ECT) electroconvulsive therapy treatment, and recommend applying to a higher center; however, they feel more comfortable during breastfeeding.^45,47^

Understandably, physicians have difficulties managing the process during the perinatal period, but this is a period when the patient especially needs physician support, follow-up, and treatment, and management should not be avoided and treatment should not be postponed.

During the interview with the patients, they should be informed that they are not helpless during this period, that different treatment options are possible during pregnancy, and that the psychiatrist will continue to care for them, follow up, and treat them during pregnancy and the postpartum period.^45^ Having a proper place to get treatment and having options is suitable for the patient.

#### Step 2: Take the Support of a Partner and Family if Possible

Spousal support is more important than ever during this period. The responsibility of taking care of the baby and the treatment should be shared. One of the most critical risks for psychiatric disorders is inadequate social support. It is possible to detect and correct possible family conflicts in interviews with the patient’s partner and, if possible, other family members.

#### Step 3: Realistic Information about using Medicine

There are fears about the use of psychotropics both in patients and health workers, specifically for malformations after the historical fact of thalidomide.

Fact: The risk of malformation for each birth is considered to be 2-4%, although it varies according to sources, regardless of drug use.^48^ If read in reverse, the probability of a woman who does not use any medication to have a baby without malformations is 96-98% (not 100%). The only risks about using medication are not congenital malformations but functional ones; neurodevelopmental issues may come over, and it may be impossible to know if it’s due to illness or the medication. There is no way to completely eliminate the risks, but treatment is possible with maximum care and safety.^9^

In addition, detailed information about the possible risks of psychiatric problems or the benefits and harms of treatment options during pregnancy and the postpartum period should be provided, and the following items should be evaluated:^45^ While using numbers when discussing risks, it should explain what they mean in real life.

- The benefits, risks, and harmful effects of treatments for psychiatric problems should be explained.
- The benefits and harms of each treatment should be explained, considering the severity of the existing psychiatric illness. Likewise, the possible risks of remaining untreated for the mother and baby should be explained.
- The possibility of the sudden emergence of mental illness symptoms during pregnancy and the postpartum period, especially in the first few weeks after birth, should be informed and noted.^44^

#### Step 4: Realistic Information about Untreated Mental Illness

Just as the harms of drug use are discussed, the possible harms of untreated mental illness should also be taken into consideration and explained to the patient.^49^

Situations such as increased risk of miscarriage during pregnancy, intrauterine growth retardation, premature birth, increased need for neonatal intensive care, attachment problems in the postnatal period, susceptibility to crime, behavioral disorders, and delay in cognitive motor development compared to peers may be observed.^49,50^ Probable risks due to untreated mental illness are summarized in Table 2.

#### Step 5: Psychotherapy and Other Non-Drug Treatment Methods

Non-drug treatment methods are possible during the perinatal period, and specifically in mild to moderate depression, psychosocial interventions should be preferred. Among these, the methods with the most evidence of effectiveness are psychotherapy, such as cognitive behavioral therapy, interpersonal psychotherapy, and stress management programs.^56-58^

In addition, it is possible to mention methods such as exercise, bright light therapy, and, when necessary, ECT, TMS, and acupuncture, according to the patient’s clinical anamnesis and symptoms.^59-61^

It is needed to know here that there is not enough evidence for methods that are thought to be completely harmless, such as herbal teas, herbal medicines, and vitamins. On the contrary, there are reports of side effects and complications.^62^ Further and more studies are needed before these methods can be considered as supportive treatment methods applicable during pregnancy.^58^

In conclusion, as with every individual, lifestyle changes/arrangement recommendations should be included in the treatment of perinatal women. Sleep quality is very important for bipolar patients, and the postpartum period is a challenging period for sleep quality.^63^ It is very important to encourage sleep, exercise, a balanced diet, and mothering (and also fathering) behaviors that develop other baby attachments for all pregnant and postpartum individuals.^58,64-66^

#### Step 6: Shared Decision-Making

A perinatal interview should be held, all information, risks, and benefits should be explained to the patient and his/her relatives, and the risks and benefits of all treatment choices should be evaluated together with the mother-to-be.^67,68^

What should be remembered at this point is that there is never a guaranteed correct answer, but predictions are possible in the light of existing literature and knowledge, and there may always be an uncontrollable unknown. Risks apply to both the disease and the medicine.^68^

Patient cooperation and co-participation are also essential in the decision-making process. After the patient and his/her relative receive the necessary information, they usually return to the physician and ask for his recommendation. At this point, the physician should clearly express his treatment recommendation, including its risks and benefits. After the patient and his/her relative give consent, stating that they accept all of these, the treatment process begins. It should not be forgotten that treatment is a biopsychosocial whole, like people and diseases.^45^

## Basic Principles of Psychopharmacologic Treatment

Along with the basic approaches of encouraging the pregnancy to be planned, not leaving the patient without treatment, and encouraging breastfeeding, the patient’s previous disorder history, how her condition was during the previous treatment/untreated periods, the current social support system, and treatment compliance should be learned. In addition, treatment should be arranged, taking into account the possible benefits and side effects of the drugs, the harms of the disease, and the current symptom severity. This chapter focuses on pharmacotherapy.

### Initiation or Continuation of Pharmacotherapy

Once the choice to initiate or continue with pharmacotherapy is made, it is crucial to determine the optimal dose to prevent inadequate treatment, together with the psychosocially supportive recommendations. The basic principles of psychopharmacotherapy during pregnancy can be listed as follows:

- The most important rule is that the mother should not be left untreated, and if pharmacological treatment is necessary, it should be used at the lowest dose that provides sufficient effectiveness. Polypharmacy should be avoided whenever possible. With each new drug added, the possibility of affecting the health of the mother and baby may increase.^9^
- Changes in treatment should be avoided whenever possible. Abruptly stopping drugs would already be a big risk. However, even during a gradual transition, multiple drug exposure occurs in crossover (the dose of the first drug is reduced while the new agent is started and increased). Moreover, there is no evidence that newly initiated treatment is worth disrupting treatment that is already going well, and treatment switching may increase the risk of relapse and recurrence. Therefore, the reuse of a drug that has been used in the past and has been beneficial should also be considered.^9^
- Untreated or inadequately treated mental illnesses should also be considered a harmful exposure for the baby. Although perinatal risks associated with the pharmacological agent should be taken into account, at the same time, one should be aware of the risks that mental illnesses may pose due to inadequate treatment.^2,9^
- If a drug known to be teratogenic is used at the beginning of pregnancy and pregnancy is suspected, the existence of pregnancy must first be ensured. It should then be explained that stopping or changing the medication will not eliminate the risk of fetal malformation. Screening for fetal abnormalities and counseling regarding the continuation of pregnancy should be provided. The need for close monitoring and the risks to the fetus if continued taking the drug should be explained. If there is uncertainty about the risks associated with certain medicines, advice should be sought from a specialist.^44^

### Titration and Ending of the Pharmacotherapy

For most medications, monitoring and dosage adjustment are achieved by monitoring symptoms. If symptoms do not improve, the dose of the medication may need to be increased. Dose increases may be required in later periods due to expected physiological changes during pregnancy, such as increased renal clearance and distribution volume and changes in the activity of drug-metabolizing enzymes. Some psychiatric medications have therapeutic medication ranges that must be monitored. It is not possible to talk about a determined therapeutic range for drugs such as selective serotonin reuptake inhibitors (SSRIs), but it should be known that drug concentrations may change during pregnancy and the postpartum period due to the reasons listed above.^69,70^

As the physiological changes that come with pregnancy end in the transition to the postpartum period, the optimal dose for psychopharmacotherapy has not been fully clarified. The postpartum transition may be a period of increased need for psychiatric support, especially for depression or anxiety disorders, and SSRIs and serotonin–norepinephrine reuptake inhibitors (SNRIs) are well-tolerated medications for this period.^71^ However, drugs whose doses and concentrations can be monitored by monitoring blood levels should be evaluated quickly after birth, and their doses should be adjusted.^2,70^

As is generally the case in the pharmacologic treatment of psychiatric disorders, sudden drug discontinuation should be avoided during pregnancy and the postpartum period. Pregnant and postpartum patients, especially those with serious mental illnesses such as bipolar disorder or psychotic disorders, are recommended to continue medication treatment.^9^ Discontinuing medications known to be effective during pregnancy or the postpartum period may increase the risk of recurrence of mental illnesses. If treatment discontinuation is planned, the risk of recurrence should be considered.^2,33^

### Pharmacotherapy During Breastfeeding

It is known that breastfeeding is vital for both mother and baby and should be encouraged^9^ It is assumed that the drug used during pregnancy and beneficial to the patient will have a similar effect during breastfeeding.^71^ However, if pharmacotherapy is started for the first time during this period, the passage of the drug into breast milk should also be taken into account.^2^ Transfer from breast milk to the baby is affected by factors such as lipid solubility, half-life, oral bioavailability, molecular weight, drug ionization, and protein binding. It is common practice to consider the relative infant dose (RID) at this point. Generally, RID rates below 10% are considered suitable for use during breastfeeding and recommended when it’s under %5.^72^ In this regard, it is necessary to make an individual-specific evaluation and create a general security profile for the treatment options.

## Psychotropic Medicines

### Antidepressants

Today, it has become a general opinion to prioritize psychotherapy as the first-line treatment for mild to moderate perinatal depression and anxiety^2,9^ In the treatment of perinatal depression or anxiety disorders, SSRIs are considered first-line medications if medication use is necessary. Although it is difficult to conduct randomized studies to evaluate the safety of antidepressant medications in the perinatal period, numerous observational studies on SSRIs support a favorable safety profile.^9^ However, in 2006, the U.S. Food and Drug Administration (FDA) published a public health advisory regarding SSRIs based on a case–control study showing an increased likelihood of neonatal persistent pulmonary hypertension (PPHT) in babies born to pregnant women who used SSRIs after the 20th week of gestation.^73^ Studies have confirmed the association between prenatal exposure to SSRIs or SNRIs and PPHN (persistent pulmonary hypretension).^74^ However, when we look at the increase in risk, it is around 1/1000 to 3/1000, which can be considered a rate that can be taken into consideration when comparing it with the risks of untreated mental illness and calculating the benefits and risks.^44^ Similar risk increase notifications should be examined by making benefits and risks calculations for other malformations and complications, and it should be taken into consideration that on the other side of the scale, there are harms of untreated mental illness.^2,44^

In the decision-making process, which medication the patient has benefited from before, what happened when he/she stopped taking the medication before, and the severity of the current symptoms are significantly critical. For individuals who have previously benefited from an antidepressant of any class (e.g., SSRI, SNRI), this medication should be the treatment of choice.^9,75^ Sertraline is often preferred due to its safety profile for people who have not used any medication in the past or for whom other medications are not effective. Escitalopram is a reasonable alternative based on efficacy and acceptability data in the general population.^76^ Because fluoxetine has a long half-life and the presence of active metabolites, it has been associated with an increased risk of neonatal adaptation syndrome and accumulation in babies, especially with its passage into milk after birth. However, if fluoxetine has previously been effective outside of pregnancy and the pregnant woman is already using this drug, it can be continued.^77^

Serotonin–norepinephrine reuptake inhibitors have been reported to be associated with an increased risk of preeclampsia in pregnancy. Additionally, a cohort study found a 1.7-fold increased risk of spontaneous miscarriage in pregnant women using SNRIs in the first trimester.^78^ However, the quality of the evidence regarding these studies is low, and as we have stated from the beginning, decision-making always involves risk-benefit analysis. Therefore, if a person has benefited from the use of an SNRI, the balance of benefits and harms favors using the previously effective drug.^2^ These antidepressants are also shown to be beneficial in rats for lowering stress in additional medical conditions, including primary psychiatric disorders.^79,80^

Brexanolone, which was recently developed for the first time as a specific drug for postpartum depression, was approved by the US Food and Drug Administration (FDA) for the treatment of postpartum depression in March 2019 and has been shown to reduce symptom severity in moderate to severe depression rapidly^81^ It has been reported that it is not necessary to stop breastfeeding because the amount of passage into milk is low.^82^ As a new treatment option, esketamine received FDA approval in 2019 for the treatment of resistant depression. ^81^ In a randomized, double-blind controlled study for postpartum depression, high-dose esketamine use was shown to reduce the frequency of depression compared to low-dose use.^83^ These studies provide hope for the future in the treatment of postpartum depression.

Melatonin and agomelatine are commonly used medications for sleep disorders and depression, but there is a need for many studies to recommend such new medicines.^50,84,85^

### Benzodiazepines

The use of benzodiazepines is occasionally observed in anxiety disorders. Long-term usage of benzodiazepines in non-pregnant individuals should also be avoided. According to the American College of Obstetricians and Gynecologists, benzodiazepines ought to be avoided unless they are utterly indispensable for the treatment of perinatal anxiety or used with caution when necessary. When benzodiazepines are prescribed, it is recommended that they be used temporarily with SSRIs, SNRIs, or psychotherapy until the anticipated response is achieved.^2^ Although earlier observational data suggested an association between benzodiazepine exposure in the first trimester and cleft lip or palate, more recent studies have not shown this association.^86^ Benzodiazepine exposure during pregnancy has been associated with neonatal sedation, decreased muscle tone, respiratory distress, and an increased need for intensive care. It is, therefore, often recommended to reduce or avoid the use of benzodiazepines during the third trimester.^78^

### Mood Stabilizers

Mood stabilizers such as lithium, valproic acid, lamotrigine, and carbamazepine are mainly used to treat bipolar disorder.^87^

Lithium has been used for bipolar disorder for years, and new mechanisms for its functions are still being discovered.^88^ It can cross the placenta and is one of the drugs with a narrow therapeutic range. If used in the perinatal period, extra care should be taken for medical conditions such as thyroid and kidney function and neurotoxicity in overdose, which normally require monitoring when using lithium. Although lithium use was thought to be associated with Ebstein anomalies in the past, recent studies have shown that keeping it in the appropriate therapeutic range does not increase the risk.^89^

Valproic acid use in the first trimester has been associated with an increased risk of neural tube defects (0.6%-2%), especially with lumbar meningomyelocele, and an increased risk of congenital malformations, specifically with high doses.^90,91^ It is possible to have planned pregnancies and reduce these risks by taking folic acid supplements before pregnancy. However, unplanned pregnancies are more common in the psychiatric population than in the general population.^92^ Even if this risk is controlled, lifelong neurodevelopmental complications may occur in the baby after the first trimester. Therefore, valproic acid is one of the treatments that should be avoided in women of childbearing age if it is possible to benefit from other medications. It is safe during breastfeeding due to the low relative infant dose.^72^

The use of lamotrigine has been shown to be safer during pregnancy and breastfeeding, especially at low doses.^93^ Although it is known that the use of carbamazepine is associated with an increased risk of congenital malformations, especially in the first trimester, this is dose-related, and its use may be possible by taking into account the increased risk and the balance of benefits and risks.^94^

### Antipsychotics

The classical pharmacological agents used to treat schizophrenia and bipolar disorder are antipsychotics. Atypical antipsychotics are preferred as alternatives to typical antipsychotics due to their better tolerability and safety profile.

Antipsychotics have been used for the treatment of psychosis for decades, and their effects on the reproductive system have been investigated.^95^ Recently, the results of an extensive cohort study on antipsychotic use have been published, and it has been reported that the use of antipsychotics in general during pregnancy is not directly related to malformation or neurodevelopmental disorder, except for the data of aripiprazole and olanzapine that require further research.^96,97^ When a major congenital anomaly was evaluated, the risk was found to be 2.7% in babies of pregnant women who were not exposed to any antipsychotics, 4.3% in pregnant women using atypical antipsychotics, and 3.1% in babies using first-generation antipsychotics. When evaluated by further analysis, it was concluded that in utero antipsychotic exposure was not significantly associated with an increased risk of malformations overall.^96^ In a large-scale cohort study, it was revealed that antipsychotic drug use during pregnancy does not have a causal relationship with the increase in the risk of neurodevelopmental disorders (NDD) such as attention deficit hyperactivity disorder and autism spectrum disorder, which may occur in the child, but the need to use antipsychotics is a predictor for NDD.^98,99^ Another large cohort study examining approximately ten thousand women with antipsychotic use and 2 million unexposed women revealed that neurodevelopmental disorders were not associated with antipsychotic use.^97^

## Conclusion

The perinatal period is a sensitive period in which psychiatric disorders are frequently observed. During pregnancy and the postpartum period, patients should not be left untreated, considering the harmful effects of the disease itself, and treatment options such as psychological interventions, lifestyle changes, and drug therapy should be evaluated. In the presence of an indication for pharmacological treatment, the initiation of appropriate pharmacotherapy is a medical responsibility with the aim of preventing patients from experiencing the adverse effects of untreated disease and delaying the progression of the disease, in the light of evidence supported by comprehensive cohort studies.

As a result, when choosing a medication during pregnancy and breastfeeding, a risk-benefit calculation should be made by knowing what the mentioned risks for untreated mental illness, drug use, and other treatment options correspond to in real life. While doing this, careful information and guidance should be provided, and a shared decision-making process should be carried out with the patient.

## Figures and Tables

**Table 1.** Basic Steps for Perinatal Interview ^45^

1	Take the responsibility of the treatment	All psychiatrist should know the basic clinical approach to perinatal women and should be ready to supply follow-up and treatment suggestions when needed.
2	Take the support of a partner and family if possible	Evaluating and motivating social support is crucial during the perinatal period.
3	Realistic information about using medicine	The benefits, risks, and harmful effects of treatments for psychiatric problems should be explained.
4	Realistic information about untreated mental illness	Just as the harms of drug use are discussed, the possible harms of untreated mental illness should also be taken into consideration and explained to the patient.
5	Psychotherapy and other non-drug treatment methods	Evidence based psychosocial treatment options are a crucial part of perinatal treatment.
6	Shared decision-making	After the receive the necessary information, with the co-participation of the patient and her relative/partner, and guidance from the doctor a shared decision-making and treatment process begins.

**Table 2.** Risks due to Untreated Mental Illness^49,51-55^

In the Mother	Pregnancy and the Postpartum	Baby-Adolescent
Impairment in self-care	Increased risk of miscarriage	Depression
Disruption in pregnancy follow-up	Intrauterine growth retardation	Autistic disorders
Nutritional deterioration	Methylation changes in fetal DNA	Growth and developmental delay
Tendency to physical diseases	Premature birth, low apgar score	Predisposition to crime
Tendency to smoke and substance use	Low birth weight	Conduct disorders
Increased risk for postpartum depression	Staying in the incubator for longer periods of time	Increased emotional, cognitive, and motor problems compared to peers
Difficulty in caring for the baby after birth	Baby sleep problems	
Suicide	Difficulty in mother-baby attachment	
